# Respiratory disease in people with major depressive disorder: A systematic review and Meta-analysis

**DOI:** 10.1192/j.eurpsy.2025.13

**Published:** 2025-02-05

**Authors:** Ana Jiménez-Peinado, David Laguna-Muñoz, María José Jaén-Moreno, Cristina Camacho-Rodríguez, Gloria Isabel del Pozo, Eduard Vieta, Javier Caballero-Villarraso, Fernando Rico-Villademoros, Fernando Sarramea

**Affiliations:** 1 Maimonides Biomedical Research Institute of Cordoba (IMIBIC), Córdoba, Spain; 2 Reina Sofia University Hospital, Córdoba, Spain; 3 Department of Morphological and Sociosanitary Science, University of Córdoba, Córdoba, Spain; 4 Department of Medicine, School of Medicine and Health Sciences, University of Barcelona (UB), Barcelona, Catalonia, Spain; 5 Bipolar and Depressive Disorders Unit, Hospìtal Clinic, Barcelona, Catalonia, Spain; 6 Institut d’Investigacions Biomèdiques August Pi i Sunyer (IDIBAPS), Barcelona, Catalonia, Spain; 7 Institute of Neurosciences (UBNeuro); 8 Centro de Investigación Biomédica en Red de Salud Mental (CIBERSAM), Instituto de Salud Carlos III, Madrid, Spain; 9 Department of Biochemistry and Molecular Biology, UGC Clinical Analyses, University of Córdoba, Córdoba, Spain; 10 Instituto de Neurociencias, Universidad de Granada, Granada, Spain

**Keywords:** asthma, chronic obstructive pulmonary disease, major depressive disorder, prevalence, respiratory conditions, treatment resistant depression

## Abstract

**Background:**

Living with major depressive disorder (MDD) reduces life expectancy, with respiratory disease being a significant threat. However, evidence on respiratory disease in this population has not yet been meta-analyzed.

**Methods:**

This meta-analysis examines respiratory disease prevalence and odds ratio (OR) in patients with MDD and treatment resistant depression (TRD). A systematic literature search was conducted, with a snowball search of reference and citation lists. Inclusion criteria covered studies in MDD and TRD patients with confirmed diagnoses of respiratory diseases (asthma, chronic obstructive pulmonary disease [COPD], pneumonia, lung cancer, and tuberculosis), comparing with a control group when possible.

**Results:**

From 4,138 retrieved articles, 15 (including 476,927 individuals with MDD, 50,680 with TRD, and 1,108,979 control group) met the inclusion criteria. In MDD patients, COPD prevalence was 9.0% (95% CI: 3.8–19.6%), asthma 8.6% (95% CI: 5.7–12.8%), and pneumonia 2.5% (95% CI: 2.2–2.9%). In TRD patients, COPD prevalence was 9.9% (95% CI: 4.2–21.9%) and asthma 10.9% (95% CI: 10.7–11.2%), but meta-analysis limited to those diseases showed no significant relative risk differences. Compared to the general population, individuals with MDD had significantly higher rates of COPD (OR 1.79, 95% CI: 1.49–2.16), even higher in younger populations (1.85 [95% CI: 1.74–1.97]) and more prevalent in women.

**Conclusions:**

This first meta-analysis on this topic shows that MDD is associated with an increased risk of respiratory illness compared to the general population. The prevalence of asthma doubles the mean described in the general population worldwide, and in COPD, women and younger people are at particular risk. Prevention policies are urgently needed.

## Introduction

Major depressive disorder (MDD) is a common disease, ranking as the third leading cause of global disease burden [1]. Nearly one in five people will experience MDD in their lifetime [2], and the one-year prevalence is very similar in high- (5.5%), middle-, and low-income countries (5.9%) [3] In addition to its impact on a person’s daily life and the huge social and economic burden it poses, depression increases the risk of all-cause mortality [4].

The link between early mortality and mental illness is an overall and transdiagnostic reality with a multidimensional origin [5]. Despite the high risk of suicide, the main cause of mortality in this population is preventable physical illness. It usually starts earlier and without timely diagnosis and treatment [5]. The gap in life expectancy with the general population keeps the same, and it is urgent to develop initiatives to understand the main risk factors [6].

An increased risk of pulmonary disease has recently been described in individuals with schizophrenia and bipolar disorder [7,8]. However, to our knowledge, this has not been meta-analyzed in individuals with MDD.

Our aim was to conduct a systematic review and meta-analysis of the five most common respiratory diseases (chronic obstructive pulmonary disease [COPD], asthma, pneumonia, lung cancer, and tuberculosis) in individuals with MDD, estimating their prevalence and odds ratio (OR) compared to the general population.

## Materials and methods

We adhered to the Preferred Reporting Items for Systematic Review and Meta-Analysis Protocol (PRISMA) [9] and the Meta-analysis of Observational Studies in Epidemiology (MOOSE) guidelines [10]. The study was registered with PROSPERO (CRD42023470972). The MOOSE checklist is presented in Supplementary Table 1.

### Information sources and searches

The search covered from inception to October 3, 2023. Two independent authors (D.L-M. and A.J-P.) searched in Pubmed, PsycINFO, and Scopus. On October 3, 2023, we extracted data using the following terms: (“major depression” OR “affective disorder” OR “mood disorder” OR “serious mental illness” OR “severe mental illness” OR “severe mental disorder”) AND (“respiratory tract diseases” OR “lung diseases” OR “asthma” OR “chronic bronchitis” OR “emphysema” OR “chronic pulmonary disease” OR “COPD” OR “chronic obstructive pulmonary disease” OR “pneumonia” OR “tuberculosis” OR “lung cancer” OR “lung neoplasms”). Moreover, our research methodology included both forward and backward reference searching: the former involved identifying articles that cited the original work post-publication, while the latter, entailed examining references or works cited within selected articles.

We included studies that: (a) focused on adult participants with a diagnosis of MDD according to established diagnostic criteria (e.g., DSM- IV [11] or ICD-10 [12]); (b) reported prevalence of respiratory disease, including asthma, COPD (including the terms chronic bronchitis and emphysema [13], pneumonia, lung cancer, or tuberculosis confirmed by means of medical diagnosis, medical records, or ICD criteria; (c) had an observational design (prospective, retrospective, or cross-sectional) and were conducted in any setting (hospital, community, or both), regardless of whether a general population control group was included; and (d) were published in an indexed, peer-reviewed journal in English.

### Outcomes

The primary outcomes were the prevalence of asthma, COPD, pneumonia, lung cancer, and tuberculosis in individuals with MDD. The secondary outcome analysis also took into consideration the comparison between individuals with major depressive disorder resistant to treatment (TRD) and those without treatment resistance (non-TRD). Moreover, we determined the combined prevalence of respiratory disease across these groups. If a study that met the inclusion criteria but did not contain sufficient data for meta-analysis, we contacted the authors to request relevant data.

### Data extraction

Two authors (A.J-P. and D.L-M.) extracted data using a predetermined data extraction form. The extracted information included the first author, country, setting, population, study design (median year, prospective, retrospective, cross-sectional), number of participants and their demographics (sex, mean age), smoking status, and antipsychotics or mood stabilizer use, as included in the article and the frequency of each respiratory disease.

### Methodological quality appraisal

The quality of each study was assessed using the Newcastle–Ottawa Scale (NOS) [14]. Points are assigned based on the selection process of cohorts (0–4 points), comparability of the cohorts (0–2 points), and identification of the exposures and the outcomes of research participants (0–3 points). We used a modified version for cross-sectional studies to adjust the evaluation [15]. The results are categorized as “Good” (7–9 points), “Fair” (5–6 points), and “Poor” (<5 points). Any disagreements were settled by discussing with another author (MJJ).

### Statistical analyses

Statistical analysis was conducted in a sequence. First, we calculated the prevalence of each specific respiratory disease among individuals with MDD, along with the 95% CI. We then compared the prevalence of each respiratory disease among individuals with MDD versus controls where available, calculating ORs together with 95% CIs. A random- effects meta-analysis was conducted. To visualize heterogeneity, prediction intervals were included in forest plots, along with calculating the I^2^ statistics for each analysis. Small study effects and publication bias were assessed by visual inspection of funnel plots and using Egger’s test when appropriate (*k* > 10) [16]. Publication bias was assessed and adjusted for with trim and fill adjusted analysis where possible. For sensitivity analyses, we calculated the subgroup differences investigating if the prevalence of each respiratory disease differed according to study design, location (Europe, North America, and Asia), and study setting. We also conducted the sensitivity analysis for the meta-analysis [17]. Likewise, the effects of potential continuous effect modifiers (mean age, median year of publication, proportion of males) were examined using meta-regression. We anticipated comparing the prevalence of respiratory diseases according to smoking status and psychotropic use, but there were insufficient data. All analyses were performed using R application [18], with packages metafor [19], meta [20], and dmetar [21].

## Results

The initial database search identified 4,133 articles after excluding duplications. Additional five studies were found through the inspection of bibliographies. In total, 4,138 articles were screened at the title and abstract level. After reviewing 92 full texts, 77 were excluded with reasons (see Supplementary Table 2). Finally, 15 unique studies met the eligibility criteria. Full details of the search results are summarized in Supplementary Figure 1.

There were 476,927 individuals with MDD with a mean age of 57.4 years (range: 45.8–77.1 years). 45.0% were male (range: 31.7–97.5%). Four studies considered asthma as their outcome [22–25]. Fourteen studies investigated COPD [23–36]. Two studies examined pneumonia [25,29]. One study considered tuberculosis as its outcome [24]. No studies provided data on lung cancer. Among TRD, there were 50,680 individuals with a mean age of 52.4 years (range: 45.9–58.9 years) and 35.3% male (range: 28.0–43.2%). In this population, two studies considered asthma as their outcome [22,23] and four studies investigated COPD [23,26,27,30]. Details of the included studies and participants are presented in Table 1 and Supplementary Table 3.

**Table 1.** Characteristics of included studies

N of study	Study	Year/s	Study design	Study location	Study setting	Data source	Respiratory condition	General population control	Risk of bias
1	Balogun (2016) [36]	2002–2012	Retrospective cohort	USA	Inpatient	University of Virginia Medical Center	COPD	Yes	Good
2	Chen (2021) [37]	2008–2011	Retrospective cohort	Taiwan	Both	Taiwan’s National Health Insurance	COPD	No	Good
3	Davydow (2016) [33]	2005–2013	Retrospective cohort	Denmark	Inpatient	Danish Civil Registration System	COPD	Yes	Good
4	DeWaters (2018) [28]	2001–2012	Retrospective cohort	USA	Inpatient	Department of Veterans Affairs (VA) Health Care System	COPD	Yes	Good
5	Lin (2019) [22]	2012–2015	Retrospective cohort	USA	Inpatient	Premier Hospital Database (Charlotte, North Carolina)	Asthma	No	Fair
6	Moser (2022) [27]	2016–2018	Retrospective cohort	Israel	Both	Maccabi Healthcare Services (MHS)	COPD	No	Good
7	Oh (2017) [31]	2003–2013	Retrospective cohort	South Korea	Outpatient	Korean National Health Insurance Service-National Sample Cohort	COPD	Yes	Good
8	Olfson (2018) [30]	2008–2014	Retrospective cohort	USA	Both	Truven Health Analytics MarketScan Medicaid Database	COPD	No	Fair
9	Pilon (2019) [26]	2010–2016	Retrospective cohort	USA	Both	Chronic Conditions Warehouse	COPD	Yes	Good
10	Rizvi (2014) [23]	2008–2009	Retrospective cohort	Canada	Outpatient	Canadian National Population Health Survey	Asthma COPD	No	Good
11	Ryu (2023) [24]	2002–2013	Retrospective cohort	South Korea	Outpatient	Korean National Health Insurance Service-National Sample Cohort	Asthma COPD Tuberculosis (previous)	Yes	Good
12	Schoepf (2014) [25]	2000–2012	Retrospective cohort	UK	Inpatient	National Health Tracing Services	Asthma COPD Pneumonia	Yes	Fair
13	Shen (2017) [32]	2000–2010	Retrospective cohort	Taiwan	Both	Taiwan National Health Research Institutes	COPD	Yes	Good
14	So-Armah (2019) [35]	2003–2015	Prospective cohort	USA	Both	U.S. Department of Veterans Affairs	COPD	Yes	Good
15	Sweer (1988) [29]	1982–1984	Retrospective cohort	USA	Inpatient	Geropsychiatry Unit (GSU) of the Western Psychiatric Insti- tute and Clinic in Pittsburgh	COPD Pneumonia	No	Good

One study was conducted in the 1980s [29], seven studies in the 2000s [24,25,28,31–33,36,37], six studies in the 2010s [22,23,26,30,34,35], and one study in the 2020s [27].

One study was prospective [35], while the remaining 14 were retrospective cohort studies. Five studies were conducted in Asia [24,27,31,32,34], two studies in Europe [25,33], and eight in North America [22,26,28–30,35,36].

Two studies included participants from the community [23,24], six from patients admitted to a hospital [22,25,28,29,33,36], and six from both community and patients admitted to hospital settings [26,27,30,32,34,35].

**Figure 1.**
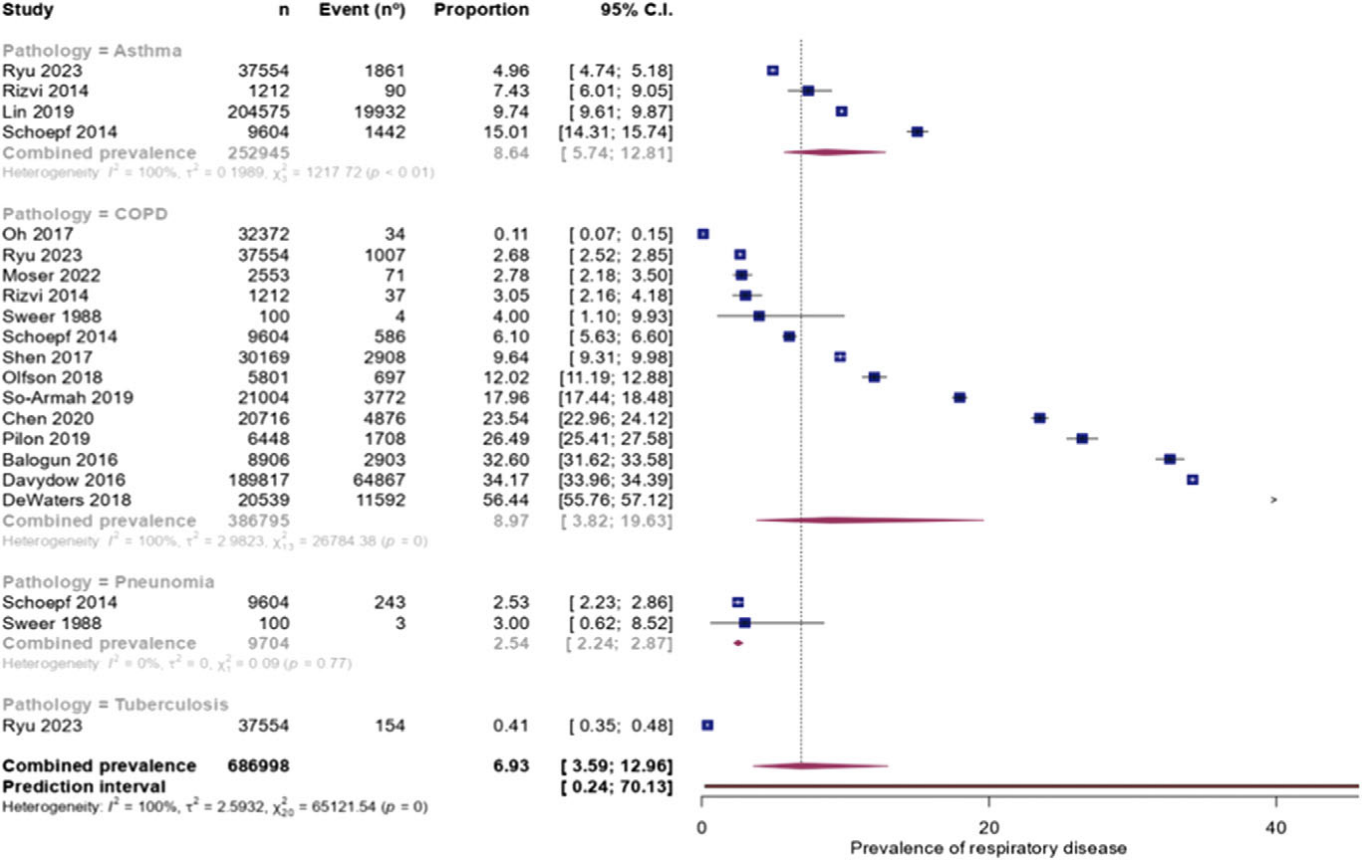
Forest plot for prevalence of respiratory disease in people with major depressive disorder.

Full details of the meta-analyses of the prevalence of respiratory diseases are presented in Table 2 and Figure 1. The funnel plot for COPD is presented in Supplementary Figure 2. The combined prevalence of respiratory disease in MDD was 6.9% (95% CI: 3.6–13.0%). There were sufficient data to meta-analyze for three conditions. Across four studies [22–25] with a total of 252,945 individuals with MDD, the prevalence of asthma was 8.6% (95% CI: 5.7–12.8%). The prevalence of COPD in 386,795 individuals with MDD was 9.0% (95% CI: 3.8–19.6%) among 14 studies [23–36]. The prevalence of pneumonia in 9,704 individuals with MDD was 2.5% (95% CI: 2.2–2.9%) among 2 studies [25,29]. Unfortunately, there was only one study of tuberculosis, making meta-analysis impossible. In Ryu et al. study, involving 37,554 individuals with MDD, the prevalence of tuberculosis was 0.4%.

**Table 2.** Prevalence of respiratory disease in people with major depressive disorder

Outcome	Study estimates	Major depressive disorder (*n*)	Prevalence (95% CI)	I^2^	Trim and fill effect size (95% CI)
Asthma	4	252,945	8.6% (95% CI: 5.7–12.8%)	99%	Not applicable
COPD	14	386,975	9.0% (95% CI: 3.8–19.6%)	100%	9.6% (3.0–27.2%)
Pneumonia	2	9,704	2.5% (95% CI: 2.2–2.9%)	0%	Not applicable

CI, confidence interval; COPD, chronic obstructive pulmonary disease

After adjusting for potential publication bias, the prevalence was slightly higher in COPD (9.6%, 95% CI: 3.0–27.2%) compared to the rest of the included diseases. After excluding outliers, the heterogeneity markedly decreased.

With respect to the TRD group, full details of the meta-analyses of the prevalence of respiratory diseases are presented in Supplementary Table 4 and Supplementary Figure 3. With a total of 50,680 individuals, the combined prevalence of respiratory disease in TRD was 9.9% (95% CI: 5.6–17.0%). There were sufficient data to meta-analyze two conditions. Across two studies [22,23] with a total of 45,390 individuals with TRD, the prevalence of asthma was 10.9% (95% CI: 10.7–11.2%). The prevalence of COPD in 5,614 individuals with TRD was 9.9% (95% CI: 4.2–21.9%) among four studies [23–36].

**Figure 2.**
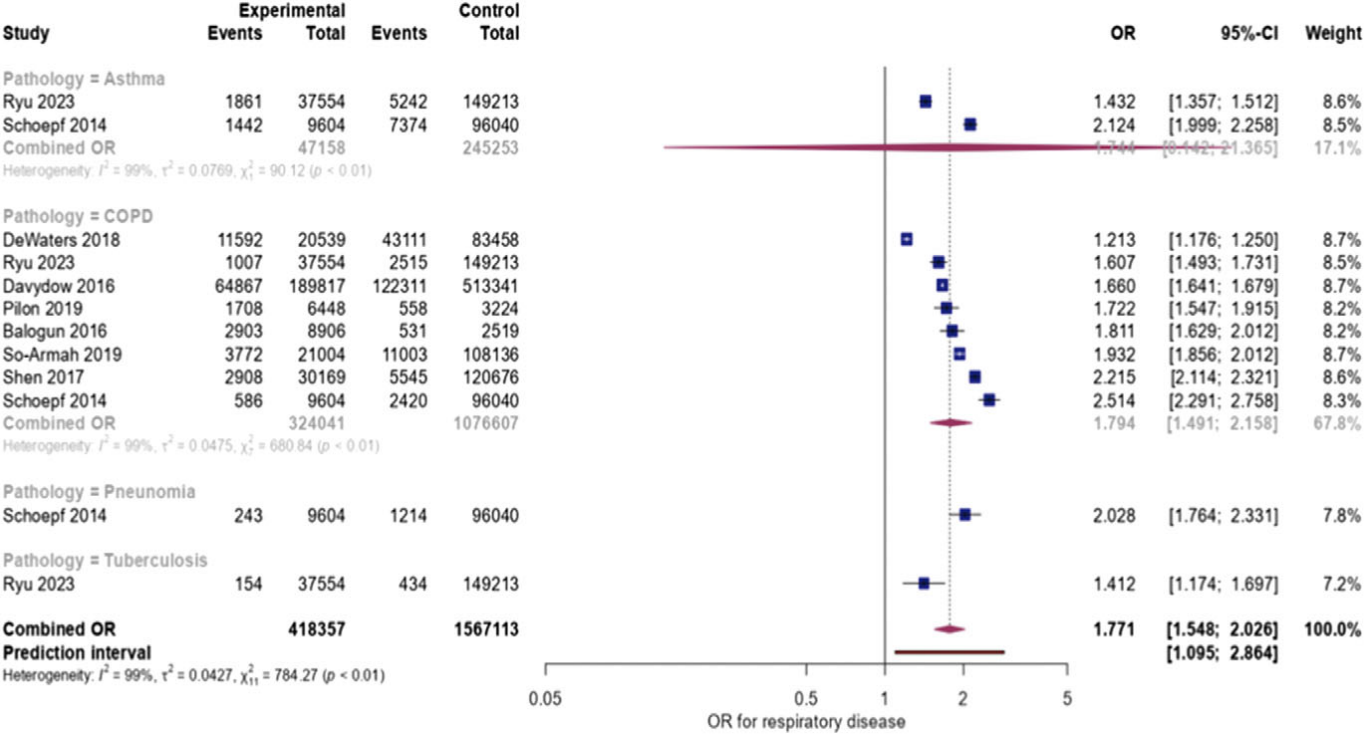
Forest plot for Odds Ratio of respiratory disease in people with major depressive disorder compared with controls.

Full details of the comparative meta-analyses are summarized in Table 3 and Figure 2. The funnel plot for COPD is presented in Supplementary Figure 4. There were sufficient data to meta-analyze two conditions: asthma (two studies [24,25]) and COPD (eight studies [24–26,28,32,33,35,36]). In those respiratory conditions, we found that individuals with MDD had higher odds of having respiratory diseases – asthma (OR 1.74, 95% CI: 0.14–21.37) and COPD (OR 1.79, 95% CI: 1.49–2.16) with no significant differences in asthma compared to individuals without MDD. After adjusting for potential publication bias using the trim and fill adjustment and after excluding outliers, the OR for COPD was still significantly higher in patients with MDD, with a value of 1.79 (79% increased risk, 95% CI = [1.49–2.15]), with more precise confidence intervals (Supplementary Figure 5). Unfortunately, there was only one tuberculosis and one pneumonia studies, being impossible to set a comparative meta-analysis. In the former study [24], the OR was 2.02 (95% CI: 1.76–2.33). In the later study [25], the OR was 1.41 (95% CI: 1.18–1.70).

**Table 3.** Odds Ratio of respiratory disease in people with major depressive disorder compared with controls

Outcome	Study estimates	Major depressive disorder (*n*)	Controls (*n*)	Odds ratio (95% CI)	I^2^
Asthma	2	47,158	245,253	1.74 (0.14–21.37)	99%
COPD	8	324,041	1,076,607	1.79 (1.45–2.16)	99%

CI, confidence interval; COPD, chronic obstructive pulmonary disease

We obtained sufficient data to perform subgroup analyses for the prevalence of COPD and asthma among individuals with MDD. Full details of the subgroup analyses of the prevalence of respiratory diseases are summarized in Supplementary Tables 5 and 6. There were no differences in the prevalence of asthma between sex, age, location, or setting. In the case of COPD, there were differences between locations. The prevalence of COPD was statistically higher in North America (16.3%, 95% CI: 7.3–32.6%) and Europe (15.5%, 95% CI: 4.2–43.7%) compared to Asia (3.0%, 95% CI: 0.6–14.1%). There were also statistically significant differences between inpatient (20.1, 95% CI: 7.4–44.4%) compared to a mixed population of inpatients and outpatients (12.9%, 95% CI: 7.0–22.4%) and outpatient group (1.0%, 95% CI: 0.2–5.4%). In meta-regression, the prevalence of COPD was higher when the percentage of women was greater (Supplementary Figure 6). The analysis revealed an adjusted sex prevalence of COPD was 9.0% (95% CI: 4.3–17.8%). There were no differences between age groups.

We found sufficient data to conduct subgroup analyses for the comparative meta-analyses for COPD (Supplementary Table 7). The likelihood ratio of COPD was greater for people with MDD in retrospective studies (OR 1.78, 95% CI: 1.43–2.21), North American studies (OR 1.64, 95% CI: 1.17–2.31), inpatient setting (OR 1.7, 95% CI: 1.10–2.80), and both inpatient and community setting (OR 1.96, 95% CI: 1.43–2.69). In the meta-regression, younger patients have an increased risk of developing COPD compared to older people (Supplementary Figure 7). The adjusted age OR for COPD was calculated at 1.85 (95% CI: 1.74–1.97). No differences were found between the sexes.

We also obtained datasets that allow for a sub-analysis, focusing on the interplay between TRD and non-TRD in asthma [22,23] and COPD [23,26,27,30] with not statistical differences in both cases (asthma OR 1.19, 95% CI: 0.00-inf; COPD OR 1.31, 95% CI: 0.01–357,238).

Finally, the mean score in the quality assessment was 6.9 out of 9 points. Twelve of the included studies (80%) received a “Good” quality and the remaining studies (20%) received a “Fair” quality assessment (Supplementary Table 8), with lower results in selection and outcome comparison. The former reflects the tendency for cases to be electronically recorded (ICD codes) instead of medical diagnoses increasing risk of selection bias, while the latter highlights the absence of additional factors or secondary outcomes.

## Discussion

This systematic review and meta-analysis of 15 studies, including 476,927 individuals with MDD, is the first to analyze the prevalence and relative risk (measured in OR) of the most frequent respiratory diseases in this population compared to the general population. The COPD likelihood was up to 79% higher in individuals with MDD, even higher in younger individuals and women. Furthermore, the prevalence of asthma in MDD was found to be twice that described in the general population worldwide.

The finding of a significant increase likelihood of COPD, even greater in the younger population, is consistent and of special concer because of the early mortality rates and the reduced life expectancy figures described in individuals with MDD [4]. The finding coincides with that recently published data in other severe mental illnesses such as schizophrenia [7] and bipolar disorder [8], with increased risks of 82 and 73%, respectively, and higher in young people and women with bipolar disorder [8].

The causes of COPD worldwide are well established, with tobacco smoking being a key factor in its development and course [38]. Individuals with a diagnosis of major depression have higher smoking rates than the general population [39] and individuals with mental illness initiate smoking at a younger age, with higher levels of dependence and heavier smoking [40]. In addition, in a more complex analysis of COPD, factors associated with depression, such as early life adversity, low educational level and low income, unhealthy diets, central obesity, sedentary lifestyles, and sub-chronic levels of inflammation, are linked to lower levels of lung function and, through this, to higher risks and early onset of COPD [41]. These factors may also explain, together with smoking severity and its early onset, the risk found in younger populations.

COPD in women is a growing public health challenge, with increasing prevalence and mortality at a faster rate than in men [42], and it is considered biologically possible that women are more vulnerable to the effect of tobacco smoking and other environmental factors [43]. It is important to highlight the fact that depression is more prevalent in women and that the relationship studied can even be bidirectional or that there is a synergist action, because just as we observed a higher prevalence of COPD in women with MDD, a higher prevalence of depression has been described in women than in men with COPD [44]. These findings in women, in the same sense of that described in younger people, translate to an opportunity for the development of preventive strategies especially in these high-risk populations.

Based on more than 250,000 individuals with MDD, the estimated prevalence of asthma (8.6%) doubles the mean described in general population worldwide of 4.3% [45]. Although the odds of asthma were higher in MDD, the statistical significance was not reached in the OR calculation. The prevalence observed is consistent with two previous meta-analyses describing a possible association, although with less restrictive inclusion criteria [46] and studying whether depression predicts the occurrence of asthma and therefore excluding those studies with depression and asthma at baseline [47]. Figures are also in the line with those recently described by in bipolar disorder [8], with a significant increased risk of asthma. To date, the predominant hypothesis is the possible association of both conditions, which could be mediated through common inflammatory mediators plus the fact that individuals with a mental illness are overexposed to a grater accumulation of health risks.

The adjusted prevalence of these conditions meta-analyzed was close to those described in the general population, being the combined prevalence of a respiratory disease in individuals with MDD of 6.9%. In COPD and adjusted for study location it was 9.0%, with higher prevalence in the USA and European studies (16.3 and 15.5%, respectively) than in Asia (3.0%), which is within the ranges described in the general world population worldwide (8–11%) [48,49]. The prevalence of pneumonia was 2.5%. There are no comprehensive global epidemiological rates for pneumonia, as these are disaggregated by the etiological pathogen (Staphylococcus, Acinetobacter, Nocardia, COVID, etc.), as evidenced in some studies [50–55]. When considering the OR and prevalence statistics, it is essential to consider the possibility of underdiagnosis and undertreatment in populations affected by a mental disorder, as highlighted in existing literature – they consult less for medical reasons, they do so in more advanced stages and with higher mortality rates [56]; therefore, there is a greater risk of underestimating differences.

Although early mortality is considered a transdiagnostic issue [5] and therefore not limited to severe mental illnesses, these conditions have been associated with the greatest reductions in life expectancy and increased severity in exposure to risk factors [57]. Therefore, we examined the subgroup of patients with TRD and found a high adjusted prevalence of respiratory disease (9.9%), even surpassing those reported in bipolar disorder [8]. Our meta-analysis, limited to data on COPD and asthma due to the fewer available studies, did not reveal significant differences in relative risks for the conditions analyzed, compared with MDD.

The results obtained are novel, occur in a highly prevalent mental disorder and are consistent with those previously described in other serious mental disorders. Moreover, they belong to a population with higher rates of early mortality and at risk of not having equal access to preventive healthcare. Nevertheless, this work is not without limitations. First, the analysis has been restricted by the lack of studies in lung cancer, and prevalence figures in TB and pneumonia have been calculated based on only one and two studies, respectively. Second, in COPD, although the heterogeneity parameters found were high, they were significantly reduced by excluding outliers, and the risk figures were maintained (1.81, 95% CI = 1.59–2.06). No publication bias was detected. Third, in the studies reviewed, the evaluation of comorbidities was retrospective and did not control for the respiratory risk variables, such as exposure to perinatal risks, socioeconomic level and general living conditions, cumulative smoking consumption, or exposure to antipsychotic treatment [58]. Fourth, as mentioned earlier, there was not enough statistical power to establish the significance of the numbers found for patients with TRD versus MDD [59]. Fifth, the absence of comprehensive smoking data across studies to explore and quantify its role as a moderator may have introduced residual confounding, reducing the precision and reliability of the meta-regression results. Last, although 80% of the studies achieved a “good” quality rating, future research should enhance methodology regarding subject selection and the assessment of all comorbidities.

## Conclusion

Individuals with MDD show a high prevalence of asthma and an increased risk than the general population of developing COPD, with women and younger people at particular risk. In a common mental disease with higher early mortality rates, equal access to treatment for smoking cessation, detection, and management of other risk factors for respiratory diseases, and early diagnosis and treatment of respiratory diseases should be promoted. The potential for underdiagnosis and undertreatment of respiratory conditions in individuals with MDD underscores the likelihood of underestimation in prevalence estimates. This is a high priority, as underreporting may significantly influence the observed relationship between MDD and the risk of respiratory diseases.

The results found in MDD, and those previously described in schizophrenia and bipolar disorder, should open a debate about the importance of monitoring respiratory function in these populations at risk by means of spirometry, an inexpensive, easy-to-handle, and non-invasive test that is easily implementable.

## Supporting information

Jiménez-Peinado et al. supplementary material Jiménez-Peinado et al. supplementary material

## Data Availability

Data are available from the corresponding author upon request.

## Supplementary material

The supplementary material for this article can be found at http://doi.org/10.1192/j.eurpsy.2025.13.
