# Supplementary material for: Respiratory disease in people with major depressive disorder: A systematic review and Meta-analysis
Source: Eur Psychiatry. 2025 Feb 5;68(1):e34. doi: 10.1192/j.eurpsy.2025.13 (PMC11883783; doi:10.1192/j.eurpsy.2025.13)
Supplement: Jiménez-Peinado et al. supplementary material [file S0924933825000136sup001.zip › Supplementary Tables.docx]

**SUPPLEMENTARY TABLES**

**Supplementary Table 1: MOOSE Checklist for Meta-Analysis of Observational studies**

| **Item No** | **Recommendation** | **Reported on Page No** |
| --- | --- | --- |
| Reporting of background should include | | |
| 1 | Problem definition | 2 |
| 2 | Hypothesis statement | 2 |
| 3 | Description of study outcome(s) | 3 |
| 4 | Type of exposure or intervention used | 3 |
| 5 | Type of study designs used | 3 |
| 6 | Study population | 3 |
| Reporting of search strategy should include | | |
| 7 | Qualifications of searchers (eg, librarians and investigators) | Title page |
| 8 | Search strategy, including time period included in the synthesis and key words | 3 |
| 9 | Effort to include all available studies, including contact with authors | 3 |
| 10 | Databases and registries searched | 3 |
| 11 | Search software used, name and version, including special features used (eg, explosion) | - |
| 12 | Use of hand searching (eg, reference lists of obtained articles) | 3 |
| 13 | List of citations located and those excluded, including justification | Table 1 and Supplementary Table 2; pages 16-17; 20-23 |
| 14 | Method of addressing articles published in languages other than English | - |
| 15 | Method of handling abstracts and unpublished studies | - |
| 16 | Description of any contact with authors | 3 |
| Reporting of methods should include | | |
| 17 | Description of relevance or appropriateness of studies assembled for assessing the hypothesis to be tested | 3-4 |
| 18 | Rationale for the selection and coding of data (eg, sound clinical principles or convenience) | 3-4 |
| 19 | Documentation of how data were classified and coded (eg, multiple raters, blinding and interrater reliability) | 3 |
| 20 | Assessment of confounding (eg, comparability of cases and controls in studies where appropriate) | - |
| 21 | Assessment of study quality, including blinding of quality assessors, stratification, or regression on possible predictors of study results | 3-4 |
| 22 | Assessment of heterogeneity | 4 |
| 23 | Description of statistical methods (eg, complete description of fixed or random effects models, justification of whether the chosen models account for predictors of study results, dose-response models, or cumulative meta-analysis) in sufficient detail to be replicated | 4 |
| 24 | Provision of appropriate tables and graphics | Table 1, 2 and supplementary material |
| Reporting of results should include | | |
| 25 | Graphic summarizing individual study estimates and overall estimates | Figure 1 and 2 |
| 26 | Table giving descriptive information for each study included | Table 1; page 16 |
| 27 | Results of sensitivity testing (eg, subgroup analysis) | 5-7 |
| 28 | Indication of statistical uncertainty of findings | 5-7 |
| Reporting of discussion should include | | |
| 29 | Quantitative assessment of bias (eg, publication bias) | 3-4 |
| 30 | Justification for exclusion (eg, exclusion of non-English language citations) | Supplementary Table 2; pages 20-23 |
| 31 | Assessment of quality of included studies | 3-4 |
| Reporting of conclusions should include | | |
| 32 | Consideration of alternative explanations for observed results | 7-9 |
| 33 | Generalization of the conclusions (ie, appropriate for the data presented and within the domain of the literature review) | 9 |
| 34 | Guidelines for future research | 9 |
| 35 | Disclosure of funding source | Title page |

**Supplementary Table 2: Reasons of the excluded studies**

| **Study** | **Reason for exclusion** |
| --- | --- |
| Abdin 2022 | No population data |
| Adekkanattu 2023 | Encompasses patients with mental illness (Major Depressive Disorder and Dysthymia) |
| Alonso 2014 | No prevalence data |
| Amital 2013 | Encompasses patients with respiratory diseases |
| Askari 2017 | Encompasses patients with mental illness (mood disorder) |
| Capo-Campos 2012 | Encompasses patients with mental illness (mood disorder) |
| Castilla-Puentes 2011 | Screening, not diagnosis |
| Choi 2018 | Encompasses patients with mental illness (Major Depressive Disorder and Bipolar disorder) |
| Cobb 2018 | Encompasses patients with mental illness (SMI) |
| Cowdery 2022 | No prevalence data |
| Daumit 2010 | Encompasses patients with mental illness (SMI) |
| Davydow 2016 | Encompasses patients with mental illness (SMI) |
| Delibas 2021 | Encompasses patients with mental illness (SMI) |
| Dornquast 2017 | Encompasses patients with mental illness (SMI) |
| Filipcic 2016 | Encompasses patients with mental illness (Major Depressive Disorder and Dysthymia) |
| Garcia-Huidoboro 2012 | No respiratory disease data |
| Gildengers 2008 | No respiratory disease data |
| Girardi 2021 | Encompasses patients with mental illness (affective disorder) |
| Goldman 2023 | Encompasses patients with mental illness (SMI) |
| Gordon 2022 | Encompasses patients with mental illness (SMI) |
| Gross 2010 | No respiratory disease data |
| Han 2023 | Encompasses patients with mental illness (mood disorder) |
| Hegeman 2017 | Encompasses patients with mental illness (Major Depressive Disorder and Dysthymia) |
| Hewer 1995 | Encompasses patients with mental illness |
| Himelhoch 2004 | Encompasses patients with mental illness (SMI) |
| Ho 2016 | Encompasses patients with respiratory disease |
| Hung 2014 | No prevalence data |
| Hyland 2021 | Encompasses patients with mental illness (SMI) |
| Jakobs 2020 | Encompasses patients with mental illness (SMI) |
| Jayatilleke 2017 | Encompasses patients with mental and respiratory disease |
| Jones 2004 | Encompasses patients with mental illness (SMI) |
| Kim 2022 | Encompasses patients with mental and respiratory disease |
| Koran 2002 | Encompasses patients with mental illness (mood disorder) |
| Laursen 2007 | No respiratory disease data |
| Lee 2016 | Encompasses patients with mental illness (SMI) |
| Lesuffleur 2019 | No prevalence data |
| Maripuu 2021 | Encompasses patients with mental illness (Psychotic disorder) |
| McCarter 2023 | No Major Depressive Disorder data |
| McQuistion 1997 | No prevalence data |
| Miller 2006 | Encompasses both patients with mental and respiratory disease |
| Mirza 2021 | Encompasses patients with respiratory disease |
| Mitchell 2013 | Encompasses patients with mental illness (SMI) |
| Moldin 1993 | Encompasses patients with respiratory disease |
| Momen 2020 | Encompasses patients with mental illness (mood disorder) |
| Momen 2022 | Encompasses patients with mental illness (mood disorder) |
| Mulugeta 2020 | No prevalence data |
| Nuyen 2005 | Encompasses patients with respiratory disease |
| Oh 2023 | Encompasses patients with respiratory disease |
| Olaya 2023 | Encompasses patients with respiratory disease |
| Osborn 2013 | No prevalence data |
| Oyon 2022 | Encompasses patients with mental illness (Major Depressive Disorder and Dysthymia) |
| Park 2021 | Encompasses patients with mental illness (mood disorder) |
| Patten 2008 | No prevalence data |
| Piatt 2010 | Encompasses patients with mental illness (SMI) |
| Polcwiartek 2021 | Encompasses patients with mental illness (SMI) |
| Räsänen 2003 | Encompasses both patients with mental and respiratory disease |
| Razzano 2015 | Encompasses patients with mental illness (SMI) |
| Reilly 2015 | Encompasses patients with mental illness (SMI) |
| Richmond-Rakerd 2021 | Encompasses patients with mental illness (SMI) |
| Romanelli 2002 | Self-report |
| Rosgen 2021 | Encompasses patients with mental illness (psychiatric disorder) |
| Salazar-Fraile 1998 | Encompasses patients with mental illness (mood disorder) |
| Schmitz 2007 | Self-report |
| Scott 2008 | Encompasses patients with mental illness (mood disorder) |
| Seminog 2013 | No prevalence data |
| Shaska 2022 | Encompasses patients with mental illness |
| Sokal 2004 | Encompasses patients with mental illness (Affective disorder) |
| Spitzer 2011 | Encompasses patients with mental illness |
| Takehara 2020 | Encompasses patients with mental illness (mood disorder) |
| Taquet 2021 | Encompasses patients with mental illness (mood disorder) |
| Teesson 2011 | Encompasses patients with mental illness (Affective disorder) |
| Temesgen 2021 | Encompasses patients with mental illness (SMI) |
| Toender 2018 | Encompasses patients with mental illness (SMI) |
| Wang 2017 | Encompasses patients with mental illness (SMI) |
| Wei 2022 | Encompasses patients with mental illness (Affective disorder) |
| Whooley 1998 | Self-report |
| Wium-Andersen 2021 | No prevalence data |

**SMI: Severe Mental Illness**

**Supplementary Table 3: Additional information of respiratory diseases studies**

| **CHRONIC OBSTRUCTIVE PULMONARY DISEASE STUDIES** | | | | | | | | | |
| --- | --- | --- | --- | --- | --- | --- | --- | --- | --- |
| N of study | Study | N of main group population | Mean age in main group | Sex in main group (male, %) | N of control population | Mean age in control group | Sex in control group (male, %) | Frequency in main group (%) | Frequency in control group (%) |
| **1** | **Balogun 2016** | 8,906 | 54.7 | 45,9 | 2,519 | 57,39 | 62,6 | 2,903 (32.6) | 531 (21.1) |
| **2** | **Chen 2020** | 20,716 | 74.4 | 37.0 | NA | NA | NA | 4,876 (23.5) | NA |
| **3** | **Davydow 2016** | 189,817 | NA | 48.5 | 513,341 | NA | 37.7 | 64,867 (34.2) | 122,311 (23.9) |
| **4** | **DeWaters 2018** | 20,539 | 77.1 | 97.5 | 83,458 | 78.0 | 98.4 | 11,592 (56.4) | 43,111 (51.7) |
| **5** | **Moser 2022** | 2,553 | NA | 35.6 | NA | NA | NA | 71 (2.8) | NA |
| **6** | **Oh 2017** | 32,372 | 45.8 | 33.4 | 32,372 | 45.8 | 33.4 | 34 (0.1) | 13,069 (40.4) |
| **7** | **Olfson 2018** | 5,801 | NA | 24.9 | NA | NA | NA | 697 (12.0) | NA |
| **8** | **Pilon 2019** | 6,448 | 58.9 | 36.2 | 3,224 | 59.0 | 36.9 | 1,708 (26.5) | 558 (17.3) |
| **9** | **Rizvi 2014** | 1,212 | NA | 31.7 | NA | NA | NA | 37 (3.1) | NA |
| **10** | **Ryu 2023** | 37,554 | NA | 34.0 | 149,213 | NA | 34.1 | 1,007 (2.7) | 2,515 (1.7) |
| **11** | **Schoepf 2014** | 9,604 | 47.6 | 39.9 | 96,040 | 47.6 | 39.9 | 586 (6.1) | 2,420 (2.5) |
| **12** | **Shen 2017** | 30,169 | 47.6 | 38.2 | 120,676 | 47.2 | 38.2 | 2,908 (9.6) | 5,545 (4.6) |
| **13** | **So-Armah 2019** | 21,004 | 49.9 | 95.6 | 108,136 | 50.4 | 97.6 | 3,772 (18.0) | 11,003 (10.2) |
| **14** | **Sweer 1988** | 100 | 72.2 | 33.0 | NA | NA | NA | 4 (4.0%) | NA |
| **ASTHMA STUDIES** | | | | | | | | | |
| N of study | Study | N of main group population | Mean age in main group | Sex in main group (male, %) | N of control population | Mean age in control group | Sex in control group (male, %) | Frequency in main group (%) | Frequency in control group (%) |
| **1** | **Lin 2019** | 90,132 | 45.9 | 43.3 | NA | NA | NA | 9,818 (10.9) | NA |
| **2** | **Rizvi 2014** | 1,212 | NA | 31.7 | NA | NA | NA | 90 (7.4) | NA |
| **3** | **Ryu 2023** | 37,554 | NA | 34.0 | 149,213 | NA | 34.1 | 1,861 (4.96) | 5,242 (3.51) |
| **4** | **Schoepf 2014** | 9,604 | 47.6 | 39.9 | 96,040 | 47.6 | 39.9 | 1,442 (15.0) | 7,374 (7.7) |
| **PNEUMONIA STUDIES** | | | | | | | | | |
| N of study | Study | N of main group population | Mean age in main group | Sex in main group (male, %) | N of control population | Mean age in control group | Sex in control group (male, %) | Frequency in main group (%) | Frequency in control group (%) |
| **1** | **Schoepf 2014** | 9,604 | 47.6 | 39.9 | 96,040 | 47.6 | 39.9 | 243 (2.5) | 1,214 (1.3) |
| **2** | **Sweer 1988** | 100 | 72.2 | 33.0 | NA | NA | NA | 3 (3.0) | NA |
| **TUBERCULOSIS STUDIES** | | | | | | | | | |
| N of study | Study | N of main group population | Mean age in main group | Sex in main group (male, %) | N of control population | Mean age in control group | Sex in control group (male, %) | Frequency in main group (%) | Frequency in control group (%) |
| **1** | **Ryu 2023** | 37,554 | NA | 34.0 | 149,213 | NA | 34.1 | 154 (0.41) | 434 (0.29) |

**Supplementary Table 4: Prevalence of respiratory disease in people with treatment resistant depression**

| **Outcome** | **Study estimates** | **Treatment resistant depression (n)** | **Prevalence (95% CI)** | **I^2^** | **Trim and fill effect size (95% CI)** |
| --- | --- | --- | --- | --- | --- |
| Asthma | 2 | 45,390 | **10.9%** (95%CI: 10.6%-11.2%) | 44% | Not applicable |
| COPD | 4 | 5,614 | **9.9%** (95%CI: 4.2%-21.9%) | 100% | Not applicable |

**CI: Confidence Interval; COPD: Chronic Obstructive Pulmonary Disease**

**Supplementary Table 5: Subgroup analysis of prevalence of asthma in people with major depressive disorder**

| Outcome | Number of study estimates | Number of participants | Prevalence (%) | 95% confidence interval | I^2^ |
| --- | --- | --- | --- | --- | --- |
| Asthma | **4** | **252,945** | **8.6** | **5.7-12.8** | **100%** |
| Region |  |  |  |  |  |
| North America | 2 | 205,787 | 9.7 | 9.6-9.9 | 86% |
| Europe | 1 | 9,604 | 15.0 | 14.3-15.7 | Not applicable |
| Asia | 1 | 37,554 | 5.0 | 4.7-5.2 | Not applicable |
| Setting |  |  |  |  |  |
| Community | 2 | 38,766 | 5.9 | 4.5-7.8 | 93% |
| Inpatient | 2 | 214,179 | 12.1 | 8.9-16.2 | 100% |

**Supplementary Table 6: Subgroup analysis of prevalence of chronic obstructive pulmonary disease in people with major depressive disorder**

| Outcome | Number of study estimates | Number of participants | Prevalence (%) | 95% confidence interval | I^2^ |
| --- | --- | --- | --- | --- | --- |
| COPD | **14** | **386,975** | **9.0** | **3.8-19.6** | **99.9%** |
| Region |  |  |  |  |  |
| North America | 7 | 64,010 | 16.3 | 7.2-32.6 | 100% |
| Europe | 2 | 199,421 | 15.5 | 4.2-46.7 | 100% |
| Asia | 5 | 123,364 | 3.0 | 0.6-14.1 | 100% |
| Setting |  |  |  |  |  |
| Community | 3 | 71,138 | 1.0 | 0.2-5.4 | 99% |
| Inpatient | 5 | 228,966 | 20.1 | 7.4-44.4 | 100% |
| Both | 6 | 86,691 | 12.9 | 7.0-22.4 | 100% |

**Supplementary Table 7: Subgroup analysis of Odds Ratio of chronic obstructive pulmonary disease in people with major depressive disorder compared to controls**

| Outcome | Study estimates | Cases | Controls | Odds ratio | 95% confident interval | I^2^  (%) |
| --- | --- | --- | --- | --- | --- | --- |
| COPD | **8** | **324,041** | **1,076,607** | **1.80** | **1.50-2.16** | **99%** |
| Design |  |  |  |  |  |  |
| Prospective | 1 | 21,004 | 108,136 | 1.93 | 1.86 – 2.01 | Not applicable |
| Retrospective | 7 | 303,037 | 968,471 | 1.78 | 1.43 – 2.21 | 99% |
| Region |  |  |  |  |  |  |
| North America | 4 | 56,897 | 197,337 | 1.64 | 1.17-2.31 | 99% |
| Europe | 2 | 199,421 | 609,381 | 2.03 | 0.14-20.05 | 99% |
| Asia | 2 | 67,723 | 269,889 | 1.01 | 0.25-14.55 | 98% |
| Setting |  |  |  |  |  |  |
| Inpatient | 4 | 228,866 | 695,358 | 1.74 | 1.08-2.80 | 99% |
| Both | 3 | 57,621 | 232,036 | 1.96 | 1.43-2.69 | 93% |
| Outpatient | 1 | 37,554 | 149,213 | 1.61 | 1.49-1.73 | Not applicable |

**Supplementary Table 8: Modified Newcastle-Ottawa Quality Assessment Scale**

| **Study** | S1 | S2 | S3 | S4 | C | E1 | E2 | E3 | Total |
| --- | --- | --- | --- | --- | --- | --- | --- | --- | --- |
| **Balogun 2016** |  | * | * | * | * | * | * | * | 7 |
| **Chen 2020** |  | * | * | * | * | * | * | * | 7 |
| **Davydow 2016** | * | * | * | * | * | * | * | * | 8 |
| **DeWaters 2018** | * | * | * | * |  | * | * | * | 7 |
| **Lin 2019** |  | * | * | * |  | * | * | * | 6 |
| **Moser 2022** | * | * | * | * |  | * | * | * | 7 |
| **Oh 2017** |  | * | * | * | * | * | * | * | 7 |
| **Olfson 2022** |  | * | * | * |  | * | * | * | 6 |
| **Pilon 2019** |  | * | * | * | * | * | * | * | 7 |
| **Rizvi 2014** | * | * | * | * |  | * | * | * | 7 |
| **Ryu 2023** |  | * | * | * | * | * | * | * | 7 |
| **Schoepf 2014** | * | * |  | * |  | * | * | * | 6 |
| **Shen 2017** |  | * | * | * | * | * | * | * | 7 |
| **So-Armah 2019** | * | * | * | * | * | * | * | * | 8 |
| **Sweer 1988** | * | * | * | * |  | * | * | * | 7 |

Newcastle Ottawa Scale for Observational Studies

- 7 or above: Good
- 5-6: Fair
- 4 or less: Poor

S1: Adequate case definition

S2: Representativeness of the cases

S3: Selection of controls

S4: Definition of controls

C: Comparability

E1: Ascertainment of the exposure

E2: Same method of ascertainment in cases and controls

E3: Non-Response rate
