# Supplementary figures and images for: Respiratory disease in people with major depressive disorder: A systematic review and Meta-analysis
Source: Eur Psychiatry. 2025 Feb 5;68(1):e34. doi: 10.1192/j.eurpsy.2025.13 (PMC11883783; doi:10.1192/j.eurpsy.2025.13)

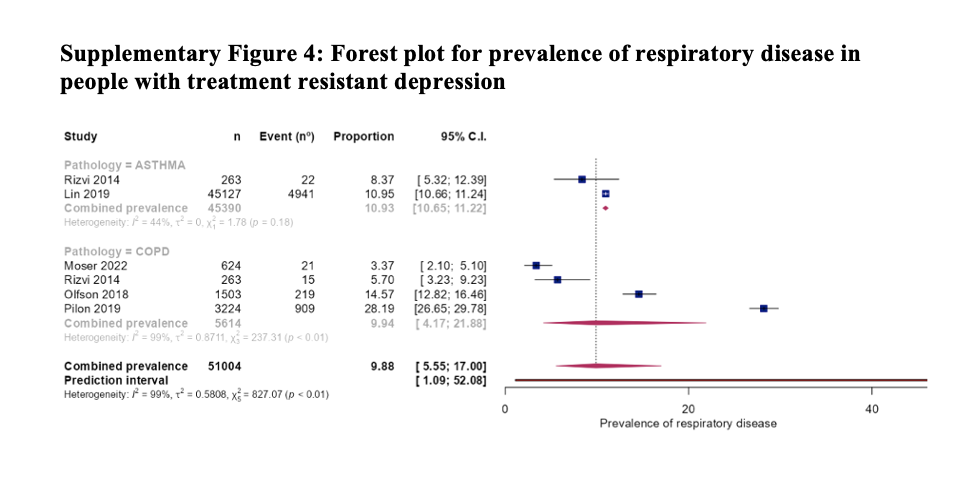

Supplement: Jiménez-Peinado et al. supplementary material [file S0924933825000136sup001.zip › Supplementary Figure 4.png]

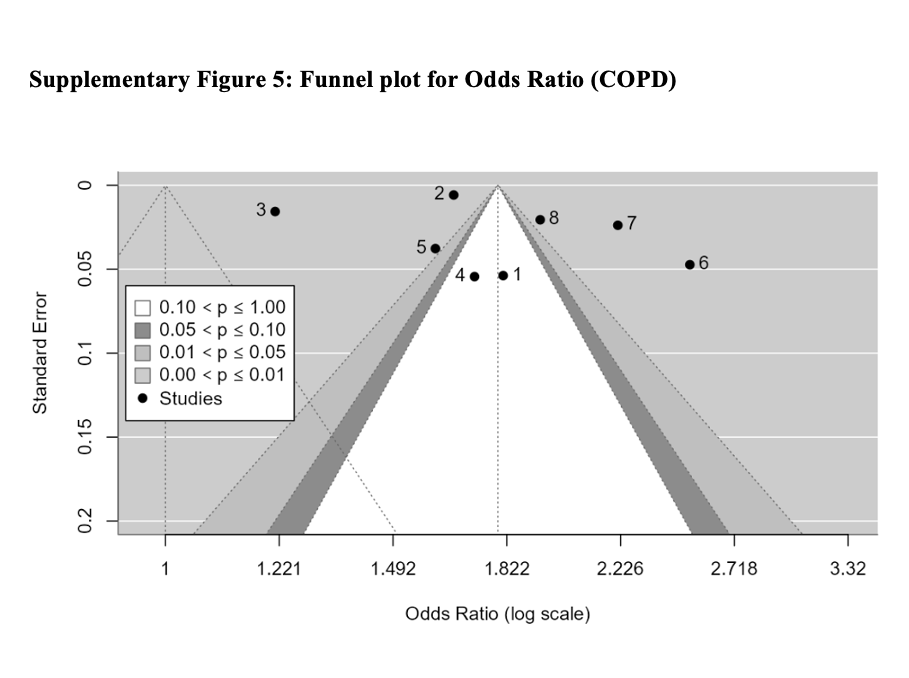

Supplement: Jiménez-Peinado et al. supplementary material [file S0924933825000136sup001.zip › Supplementary Figure 5.png]

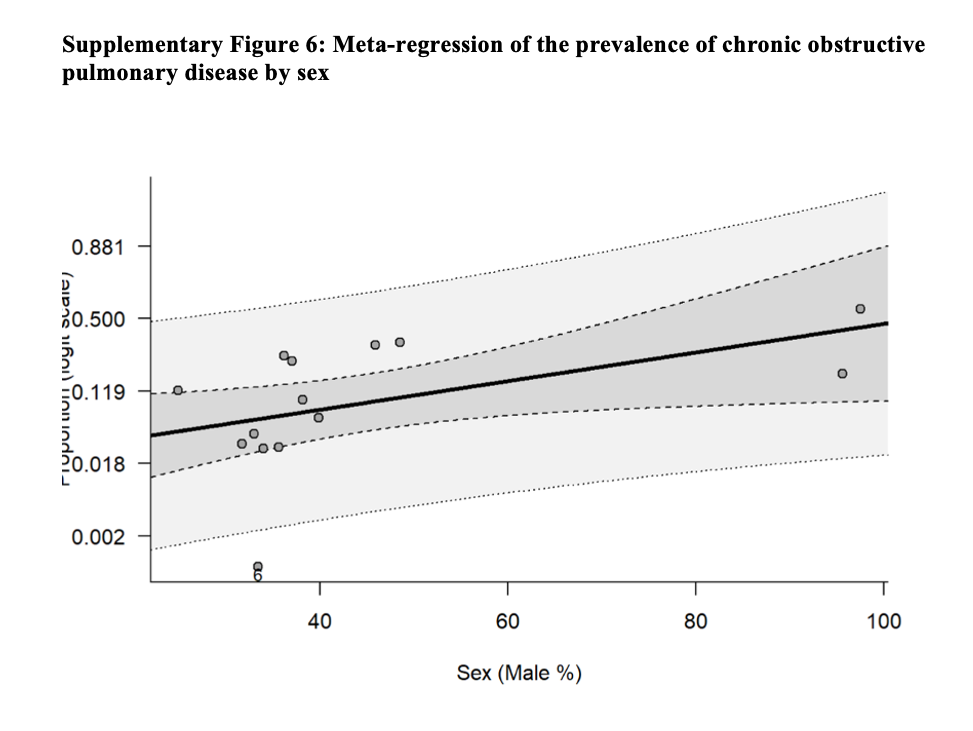

Supplement: Jiménez-Peinado et al. supplementary material [file S0924933825000136sup001.zip › Supplementary Figure 6.png]

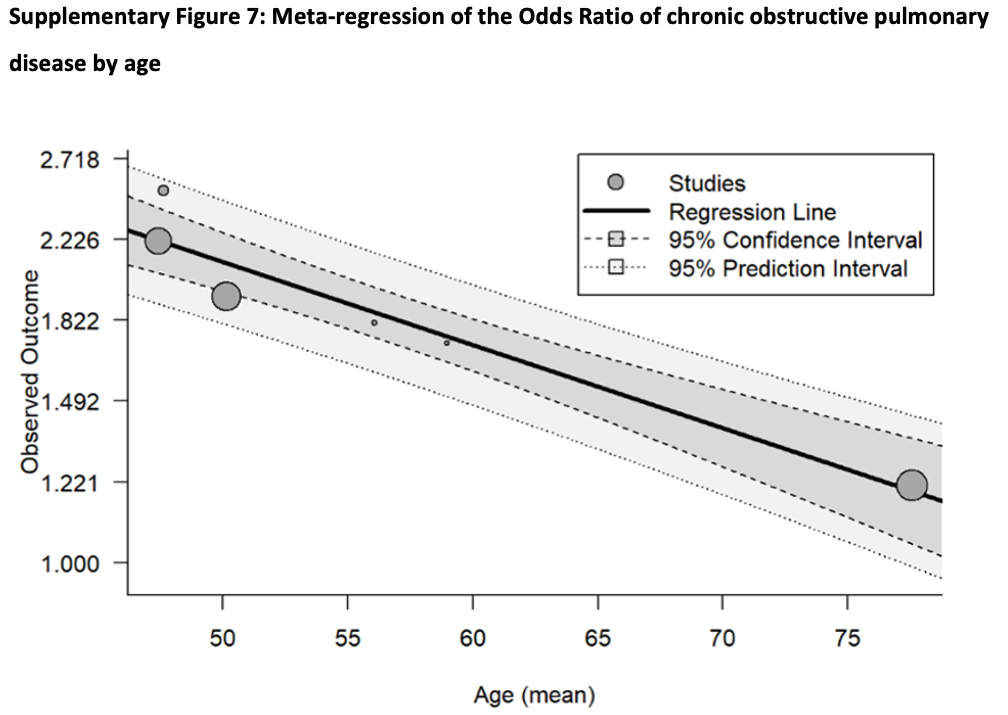

Supplement: Jiménez-Peinado et al. supplementary material [file S0924933825000136sup001.zip › Supplementary Figure 7.png]

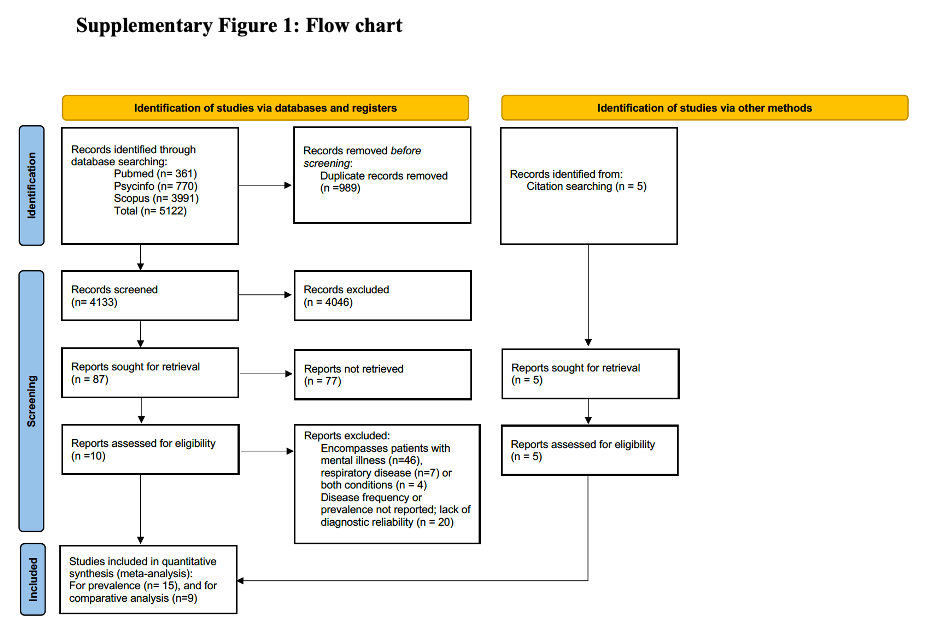

Supplement: Jiménez-Peinado et al. supplementary material [file S0924933825000136sup001.zip › Supplementary Figure 1.png]

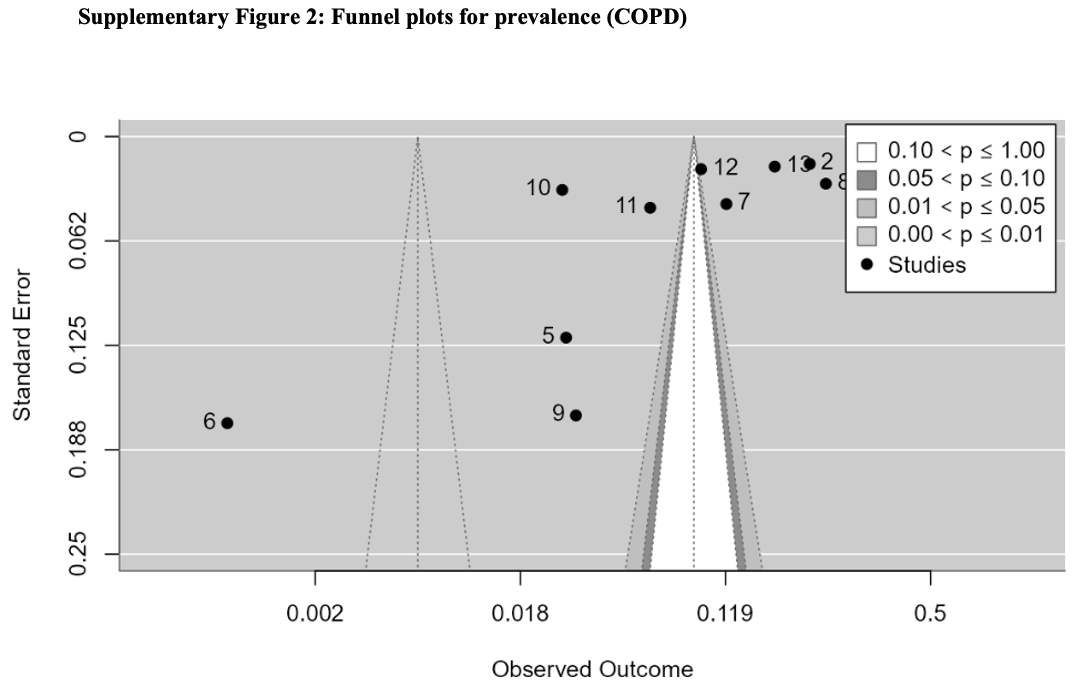

Supplement: Jiménez-Peinado et al. supplementary material [file S0924933825000136sup001.zip › Supplementary Figure 2.png]

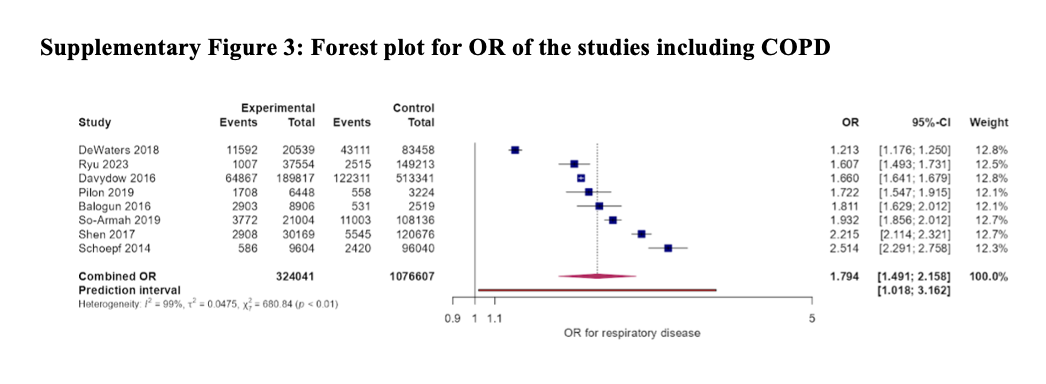

Supplement: Jiménez-Peinado et al. supplementary material [file S0924933825000136sup001.zip › Supplementary Figure 3.png]
